# Report on the 19th international symposium on geo-disaster reduction/high-level academic forum on disaster mitigation and integrated risk defense on the Plateau, 12–15 July 2021, Xining, China

**DOI:** 10.1186/s40677-021-00197-9

**Published:** 2021-10-05

**Authors:** Fenggui Liu, Guolong Zhu, Fawu Wang

**Affiliations:** 1 Research Center of Plateau Disaster Reduction and Emergency Management, Chengxi District, Qinghai Normal University/Academy of Plateau Science and Sustainable, People’s Government of Qinghai Province & Beijing Normal University, China. No. 38 Wusi Xi Road, Xining City, Qinghai Province China; 2 College of Civil Engineering, Tongji University, 1239 Siping Road, Shanghai, 20092 China

## Abstract

The joint event of 19th International Symposium on Geo-disaster Reduction (19ISGdR) and High-Level Academic Forum on Disaster Mitigation and Integrated Risk Defense on the Plateau was held on 11–15 July in Xining, Qinghai Province, China, focusing on the theme of “Geological disaster and integrated risk defense”. This event consisted of keynote lectures, invited lectures, and Youth forum, which provided a platform for scientists, industrial professionals and young scholars to share their research progress and exchange novel ideas on geo-disaster reduction in a hybrid way of offline and online. A post-symposium field trip for three days was also conducted in the joint area between Qinghai-Tibet plateau and Loess plateau.

## Introduction

The 19ISGdR/High-Level Academic Forum on Disaster Mitigation and Integrated Risk Defense on the Plateau event was jointly organized by the International Consortium on Geo-disaster Reduction (ICGdR), Qinghai Normal University/Academy of Plateau Science and Sustainable, People’s Government of Qinghai Province, Beijing Normal University, Tongji University, Emergency Management Department of Qinghai Province, and International Institute for Space Planning and Sustainable Development.

The academic committee of this event was led by three co-chairs, Peijun Shi (President of Qinghai Normal University/Academy of Plateau Science and Sustainability, China), Fawu Wang (ICGdR President/Tongji University, China), and Zhenjiang Shen (ICSPSD President/ Kanazawa University, Japan).

The organizing committee of this event was led by three Co-Chairs, Fenggui Liu (Academy of Plateau Science and Sustainability, China/School of Geographic Sciences, Qinghai Normal University, China), Ningsheng Chen (Institute of Mountain Hazards and Environment, CAS) and Qiang Zhang (Beijing Normal University, China).

The joint event was focusing on the theme of “Geological disaster and integrated risk defense” with two stages, i.e., the online stage on 2nd July, and the onsite symposium on 11–12 July, followed by a field trip in north part of Qinghai Province on 13–15 July. This event attracted more than 100 scientists, engineers, young scholars from 11 countries who provided more than 50 academic presentations on five topics, including: (1) Formative mechanism and the environment of geological disasters; (2) Recognition and early warning technology of geological disasters; (3) Risk assessment and prevention planning of geological disasters; (4) Geological disaster control and geological environment protection; and (5) Practice of geological disaster prevention and mitigation and regional sustainable development.

The presentations were carried out in one Plenary Session of keynote lectures and three Parallel Sessions including Invited Lectures and Youth Forum. As usual, Youth Forum was organized to provide a platform for the young scholars to present and share their research progress and novel ideas.

## Online part of the event

The online part was conducted on 2 July using the cloud meeting software. Before the academic presentation, the 2021 General Board of Members meeting of International Consortium on Geo-disaster Reduction (2021 GBM-ICGdR) was conducted. The meeting was focusing on the annual development and further plans of the consortium. In the academic presentation, Prof. Sabatino Cuomo (University of Salerno, Italy) presented the keynote lecture titled as “Protection measures against flow-like landslides”, and Prof. Guoquan Wang (University of Houston, USA) presented the keynote lecture titled as “Stable geodetic reference frames for long-term geological hazards monitoring in China”.

The online Youth Forum had 5 young scholars presented their recent research. They are Dr. Yuko Serikawa (National Institute of Technology, Fukui College, Japan), Mr. Takumi Murata (Kanazawa University, Japan), Ms. Angela Di Perna (University of Salerno, Italy), Dr. Akira Murata (Kanazawa University, Japan) and Mr. Daiki Nishiyama (Kanazawa University, Japan).

At last, Prof. Fawu Wang, the president of ICGdR made a brief conclusion and expressed the thanks to all the board members and young scholars to participate this activity in the special online form during the worldwide COVID-19 pandemic occasion.

## Onsite part of the event

The onsite part of the event includes one day symposium on 12 July and three days post-symposium field trip on 13–15 July. Below are the details.

### Plenary session

#### Opening ceremony

The opening ceremony was chaired by Prof. Ningsheng Chen, researcher from the Institute of Mountain Hazards and Environment, Chinese Academy of Sciences (CAS) (Fig. 1a). Prof. Guangchao Cao, the vice president of Qinghai Normal University, expressed warm welcome to all the attendance on behalf of the host at first, and then presented a brief introduction on the university development and recent status about the disaster mitigation and the integrated risk defense on the plateau, and closed his speech with the best wishes for a successful event and a bright future of ICGdR (Fig. 1b). Then, Prof. Fawu Wang, the president of ICGdR, expressed his appreciation to the organizing committee and all the working staffs. He briefly introduced the establishment and development history of ICGdR, from its forerunner, original missions, structures and members, to the impressive and exciting activity of the consortium during the last decade (Fig. 1c).

**1:**
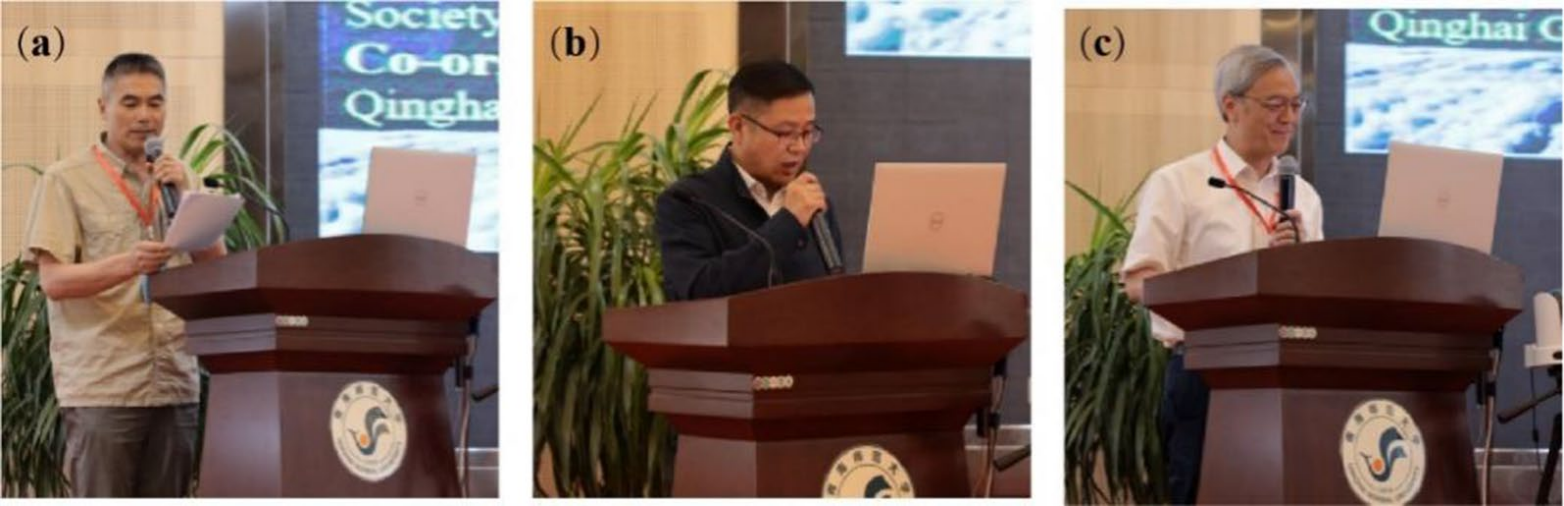
Opening ceremony. **a** chaired by Prof. Ningsheng Chen, **b** welcome speech from Prof. Guangchao Cao, vice president of Qinghai Normal University, and **c** opening speech by Prof. Fawu Wang, the president of ICGdR

At the end of the opening ceremony, Prof. Fawu Wang presented the membership certificate to the new members of ICGdR (Fig. 2) and best paper award to the winner of 2020 Best Paper Award of Geoenvironmental Disasters, the official journal of ICGdR (Fig. 3). The new memberships included: Chong Xu and Xiwei Xu (National Institute of Natural Hazards, Ministry of Emergency Management of China), Shimei Wang (China Three Gorges University), Yonggang Jia (Ocean University of China), Fenggui Liu (Qinghai Normal University), Yifei Cui (Department of Hydraulic Engineering, Tsinghua University, China), as well as individual members, Yonggui Chen and Wuwei Mao (Tongji University, China), Zili Dai (Shanghai University, China), Ningsheng Chen (Institute of Mountain Hazards and Environment, Chinese Academy of Sciences), and Jidong Teng (Central South University, China).

**2:**
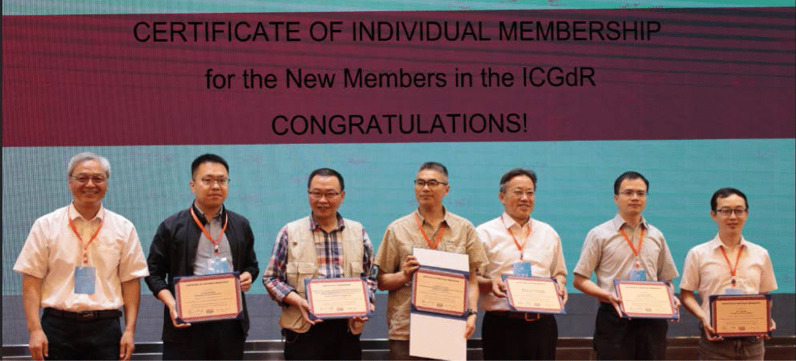
ICGdR President Fawu Wang bestowed the new ICGdR membership certificates to the members attended the event onsite

**3:**
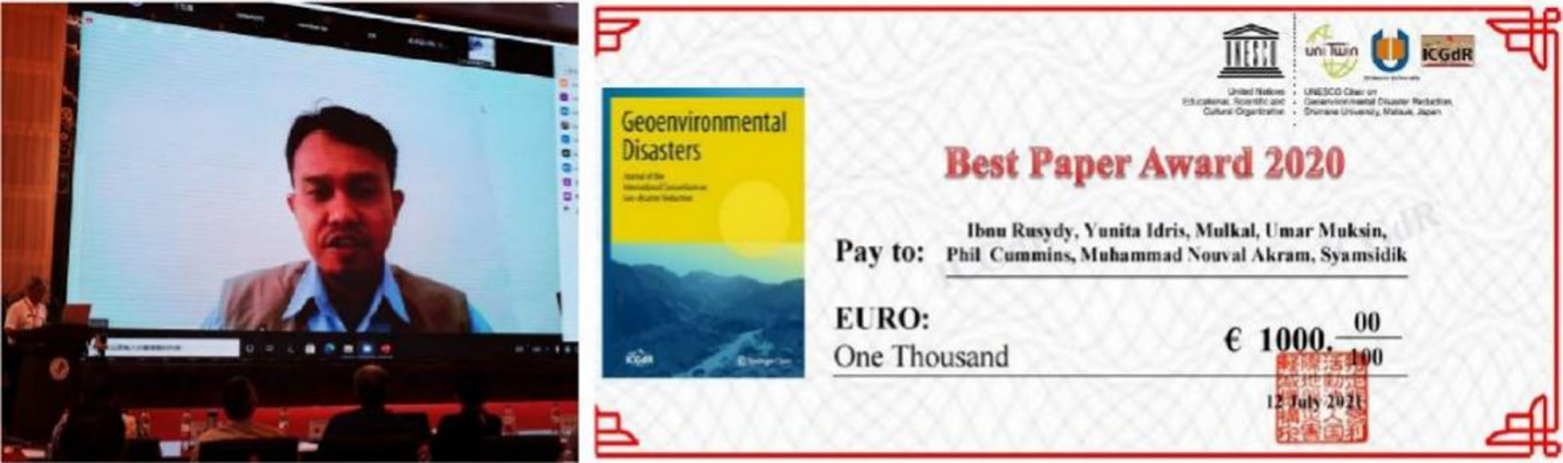
Best Paper Award 2020 of the ICGdR official journal: Geo-environmental Disaster (Dr. Rusydy’s online connection, representing all of the authorships)

The Best Paper Award 2020 of Geoenvironmental Disasters was bestowed to the paper “Shallow crustal earthquake models, damage, and loss predictions in Banda Aceh, Indonesia” by Ibnu Rusydy et al. A cheque, worth 1,000 Euro, were awarded to the authors online, and Dr. Ibnu Rusydy received the award online representing all of the authors of the paper (Fig. 3).

Finally, the opening ceremony was ended up with a group photo as shown in Fig. 4.

**4:**
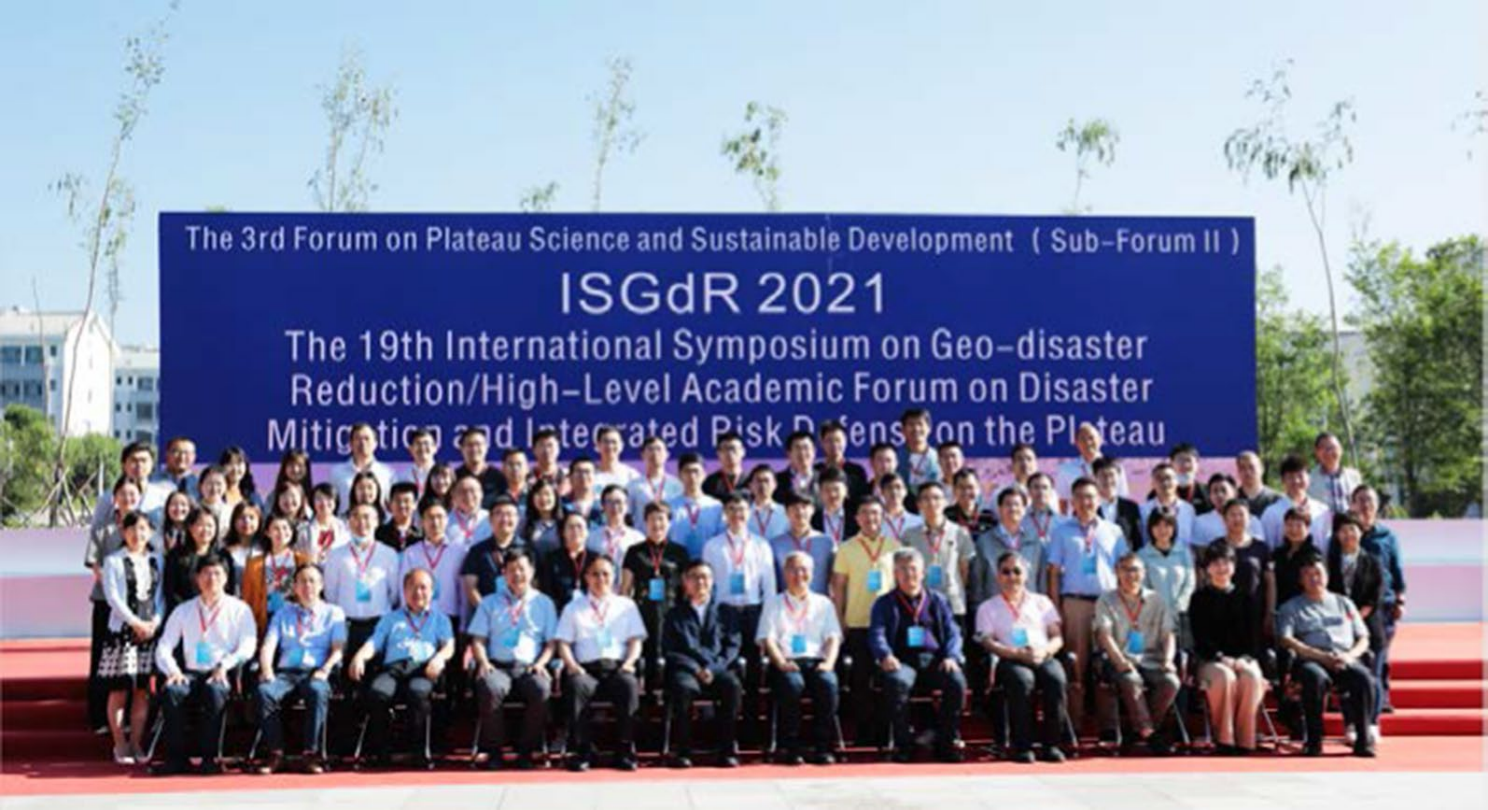
Group photo of participants to the ISGdR 2021, 19ISGdR/High-Level Academic Forum on Disaster Mitigation and Integrated Risk Defense on the Plateau

#### Keynote lectures

The ISGdR 2021 arranged 6 Keynote Lectures. The keynote lectures covered various topics related to the theme of this symposium, including geology and hydrogeology background of geo-disasters, mechanism analysis of geo-disasters, investigation methods, prevention and control measures, and so on (Fig. 5). The details of the keynote lectures, including topic titles, presenters and their affiliations, were summarized in Table 1.

**5:**
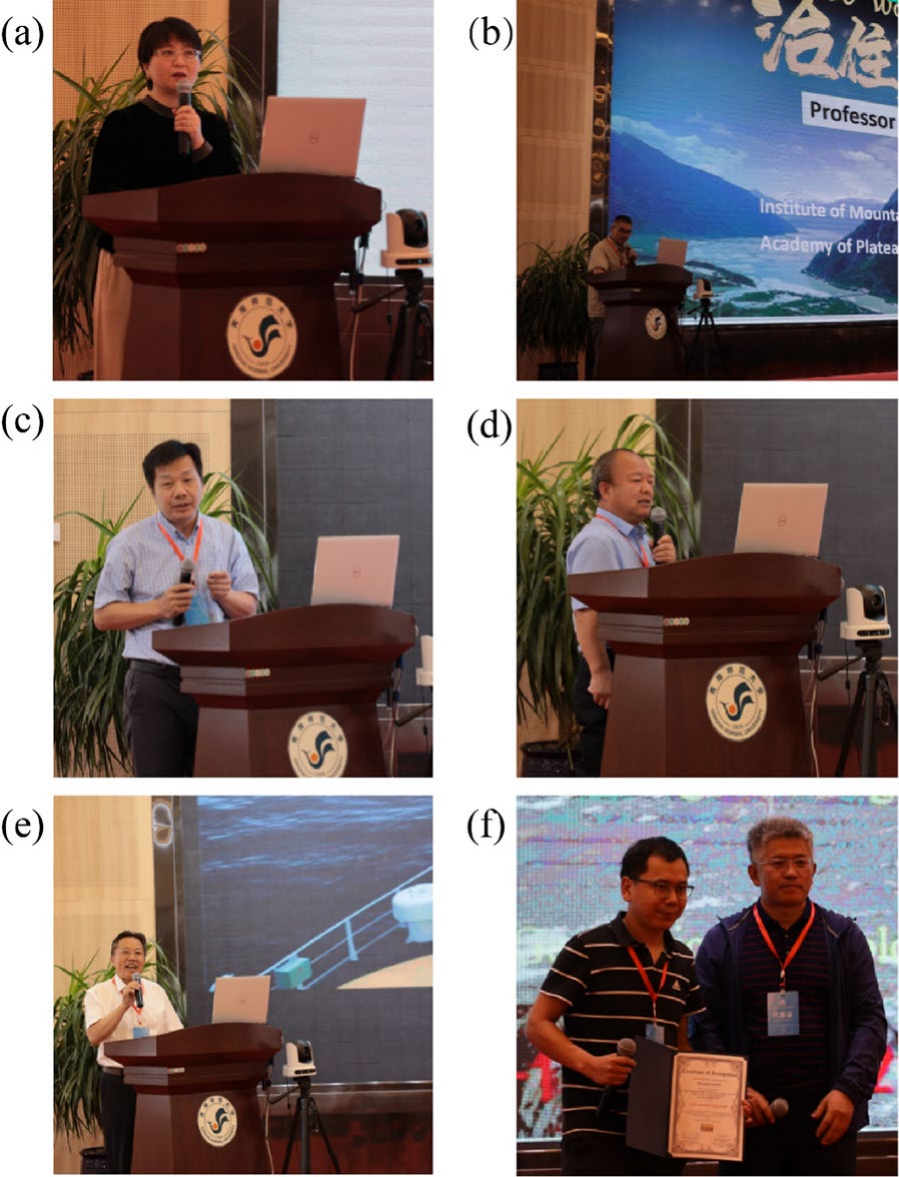
Photos of Keynote Lectures. **a** Prof. Fan Zhang, **b** Prof. Ningsheng Chen, **c** Prof. Huabin Wang, **d** Prof. Tonglu Li, **e** Prof. Yonggang Jia, **d** Chairman shows the certification for Prof. Shengwen Qi (online).

**Table 1:** List of the presentations in keynote lecture session

No.	Title	Presenter	Affiliation
K-1	Variation of sediment transport of rivers in Qinghai-Tibet Plateau	Prof. Fan Zhang	Institute of Tibetan Plateau Research, CAS
K-2	Formation and Prevention of Mountainous Disasters	Prof. Ningsheng Chen	Institute of Mountain and Environment, CAS
K-3	Microstructure evolution and mechanical properties of granite residual soil considering the free iron oxide	Prof. Huabin Wang	Huazhong University of Science and Technology, China
K-4	Investigation for the failure mechanism of the Shanyang catastrophic rock landslide	Prof. Tonglu Li	Chang’an University, China
K-5	In-situ surveying equipment of engineering geology in complex deep sea (SEEGeo)	Prof. Yonggang Jia	Ocean University of China
K-6	Experimental study on dynamic response of rock slope under strong earthquake by large shaking table	Prof. Shengwen Qi	Institute of Geology and Geophysics, CAS

#### Parallel sessions

For a further discussion on the issues of geological disaster cases, regional feathers, investigation, mechanism, prevention and etc., the symposium set three parallel sessions. There were 10 invited lectures and more than 20 presentations in the Youth Forum on the afternoon of 12 July. The information of the invited lectures was listed in Table 2.

**Table 2:** List of the invited lectures

No.	Title	Presenter	Affiliation
L-1	Evolution process investigation on the landslide dam formation using a numerical and experimental scheme	Prof. Tingkai Nian	Dalian University of Technology
L-2	Rockfall size prediction of gently inclined red-bed in Sichuan Basin	Prof. Xiaoyan Zhao	Southwest Jiaotong University
L-3	Evolution mechanism of solid-fluid phase transition in the whole process of sand flow liquefaction	Prof. Bin Ye	Tongji University
L-4	Investigation of the spatial variability of loess physical-mechanical properties	Prof. Ling Xu	Xi’an Jiaotong University
L-5	The potential application of the Granular Jamming theory in geohazards reduction	Prof. Hu Zheng	Tongji University
L-6	Study on the deformation characteristics of the giant Xiongba ancient landslide along the Jinsha River, Tibet, China	Prof. Changbao Guo	Institute of Geomechanics, CAGS
L-7	Cooling effect of the intensified ventilation composite embankment in permafrost regions	Prof. Hong Sun	Shanghai Jiao Tong University
L-8	Reconstruction of ground motion amplification based on an SMASS array site	Prof. Ping Li	China Institute of Disaster Prevention
L-9	Study on the mechanical mechanism and application of plant ecological protection in the Northeastern part of the Qinghai-Tibet plateau	Prof. Xiasong Hu	Qinghai University, China

The parallel sessions provided platforms for experts and young scholars more in-depth exchanges and discussions. The young scholars got great opportunity to summarize and share their ideas and research progress, and got lots of encouragement and advices during the discussion. Figure 6 showed the presenters in the invited lecture session.

**6:**
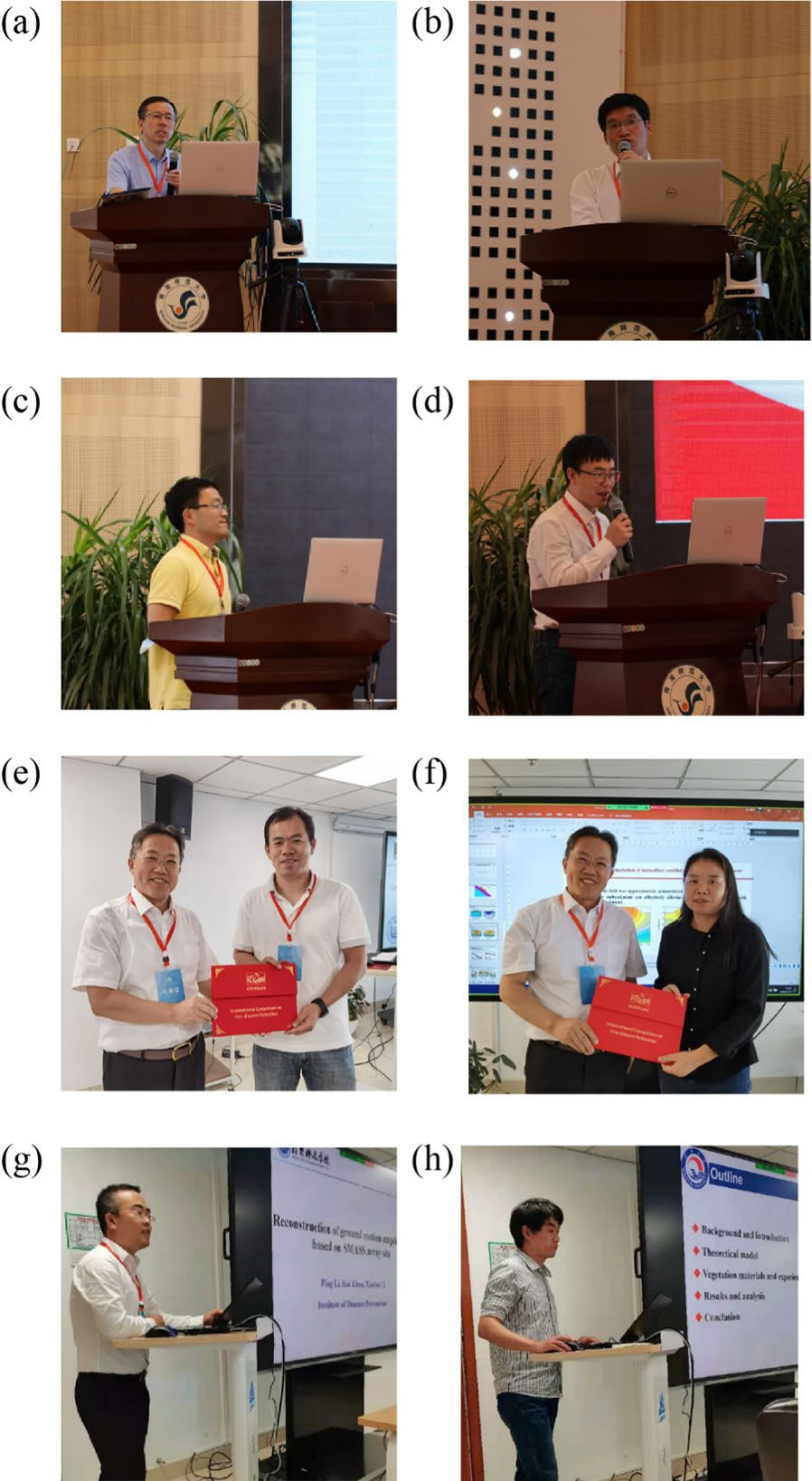
Plenary lectures in the parallel sessions presented by **a** Prof. Tingkai Nian, **b** Prof. Xiaoyan Zhao, **c** Prof. Bin Ye, **d** Prof. Ling Xu, **e** Prof. Hu Zheng, **f** Prof. Hong Sun, **g** Prof. Ping Li, **h** proxy of Prof. Xiasong Hu

#### Closing ceremony

As a prospect, the Promising Young Scientist Awards of the 19th ISGdR were announced during the closing ceremony. Prof. Fawu Wang and representatives of ICGdR Board gave the certifications to the youth scholars (Fig. 7).

**7:**
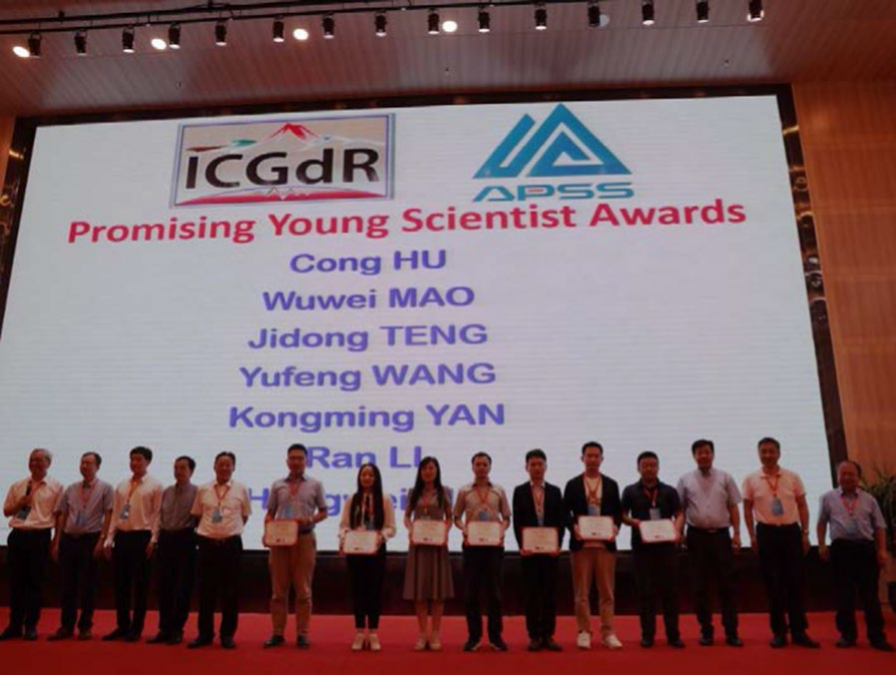
Prof. Fawu Wang and representatives of ICGdR Board bestowing the Promising Young Scientist certificates to the winners during the closing ceremony

At last, Prof. Fawu Wang, on behalf of ICGdR, gave a closing speech and expressed sincere appreciation to the organizer and co-organizers for this special symposium during the worldwide COVID-19 pandemic period and sincerely looked forward to meeting all distinguished guests in the 20th ISGdR, which will be held in Kanazawa, Japan in August 2022.

### Post-Symposium Field trip

Followed the onsite symposium, a field trip to the north part of Qinghai Province was conducted on 13–15 July. 42 participants attended the field trip. This trip focused on the geological and topographical feathers at the boundary between Qinghai-Tibet Plateau and Loess Plateau. During the field trip, the group visited the Danxia landform near Qilian County in the Beihai Tibetan Autonomous Prefecture (Fig. 8). The red layered sedimentation could be observed, and the layers were approximately below a certain height, where we could imagine the original terrain in the surrounding mountains and peaks (Figs. 9 and 10). Based on the above phenomena, it could be considered that the lacustrine deposits distributed in this Danxia landform area. After deposition, it appears red due to the appropriate oxidation environment. It could be speculated that large-scaled landslides occurred here, resulted in a formation of barrier lake and lacustrine sedimentation.

**8:**
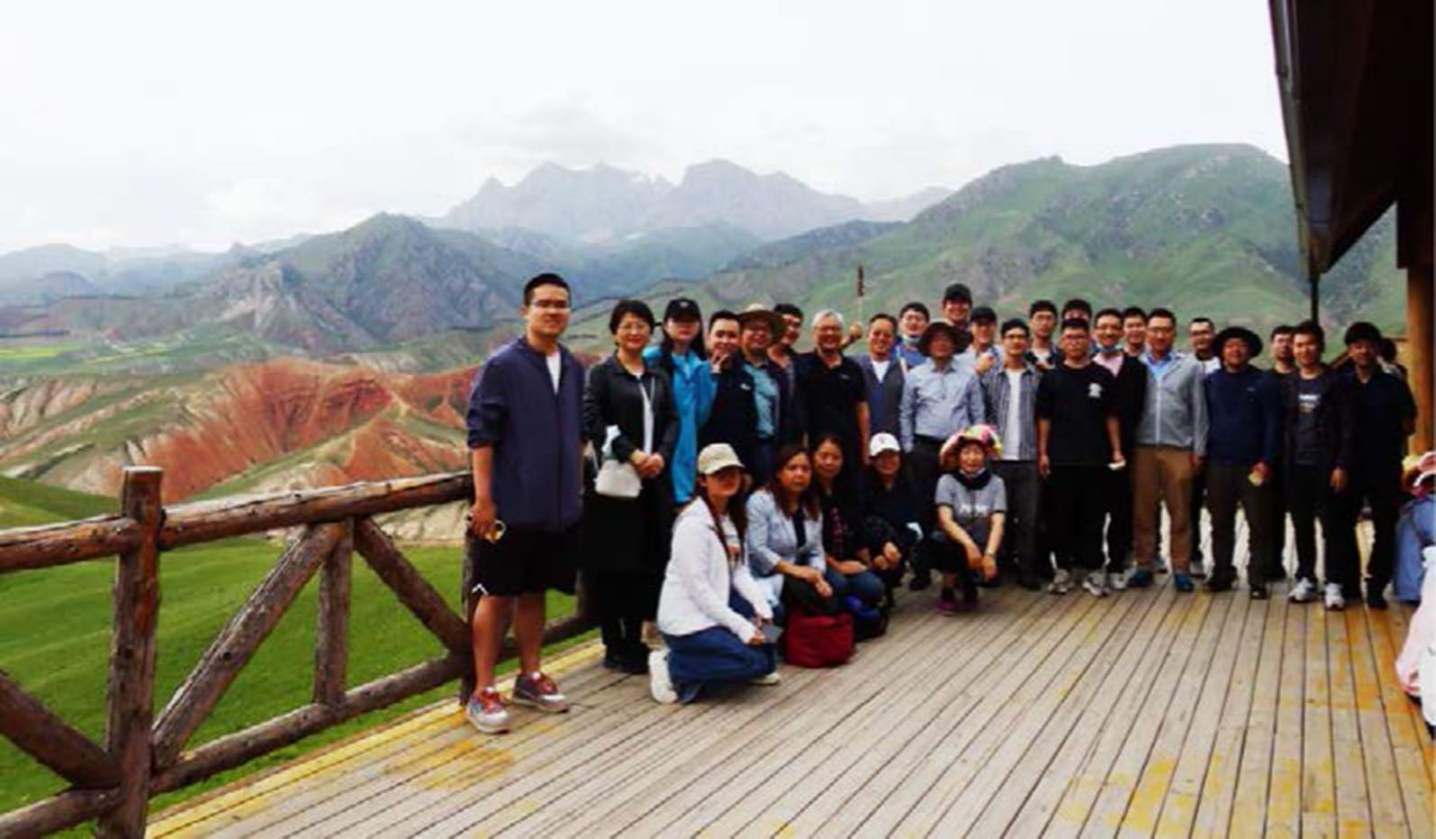
Group photo of field trip participants at the Danxia landform site near Qilian County

**9:**
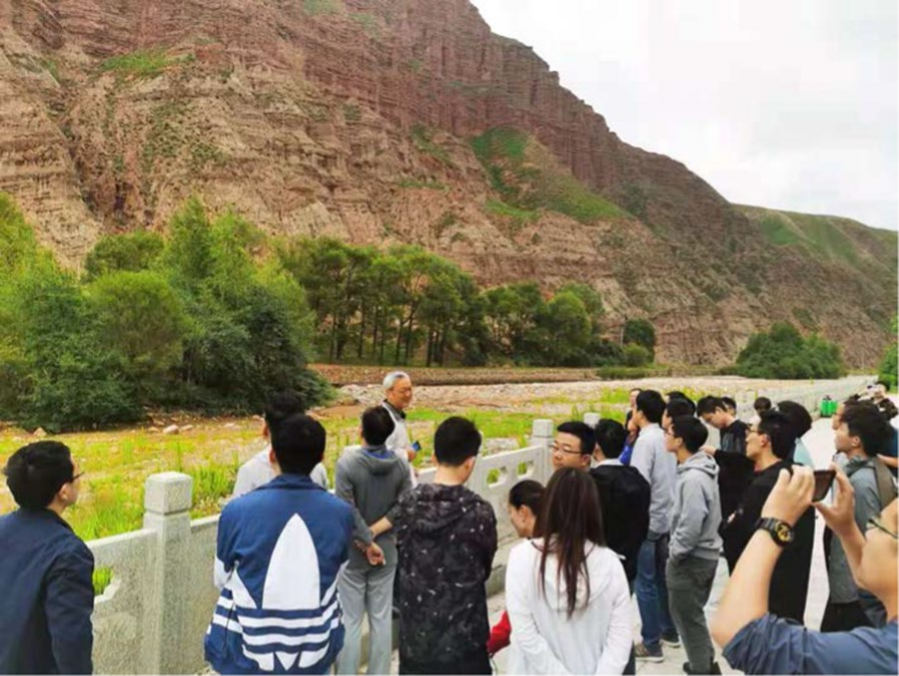
On-site explanation and discussion during the field trip

**10:**
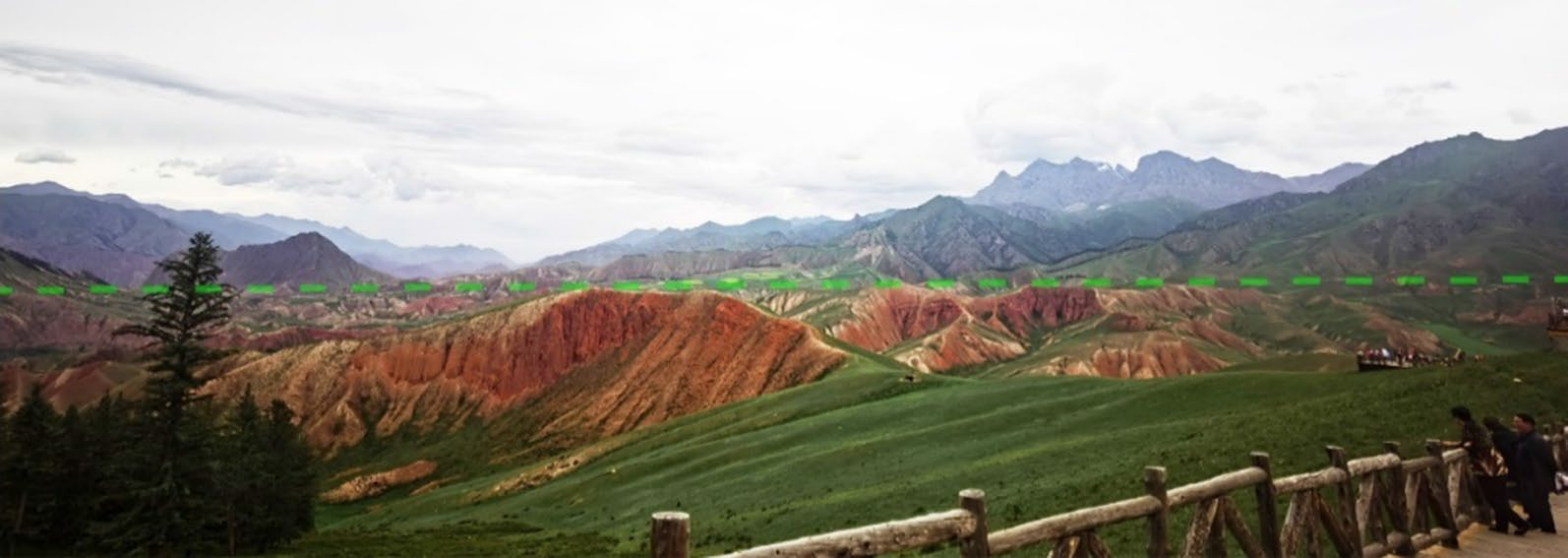
The layered sedimentation and lacustrine deposits distributed in the Danxia landform area

In Gangca County located on the Qinghai-Tibet Plateau landform, several Geo-disaster sites were observed and visited by the group. As shown in the photos during the trip, one of the landslides from the source area divided into two branches in the accumulation area (Fig. 11a). Figure 11b showed a typical circular landslide with a relatively intact landslide bench, while, Fig. 11c showed debris slides with a short sliding distance comparing to a typical debris flow. Besides, a probable debris flow site was observed on the Qinghai-Tibet Plateau landform. Since there were large rocks with random distribution and poor roundness on both sidewalls of the gully (see Fig. 12), it could be judged that this area was the accumulation fan of an old debris flow, and it might be impacted by another debris flow formed in the current gully.

**11:**
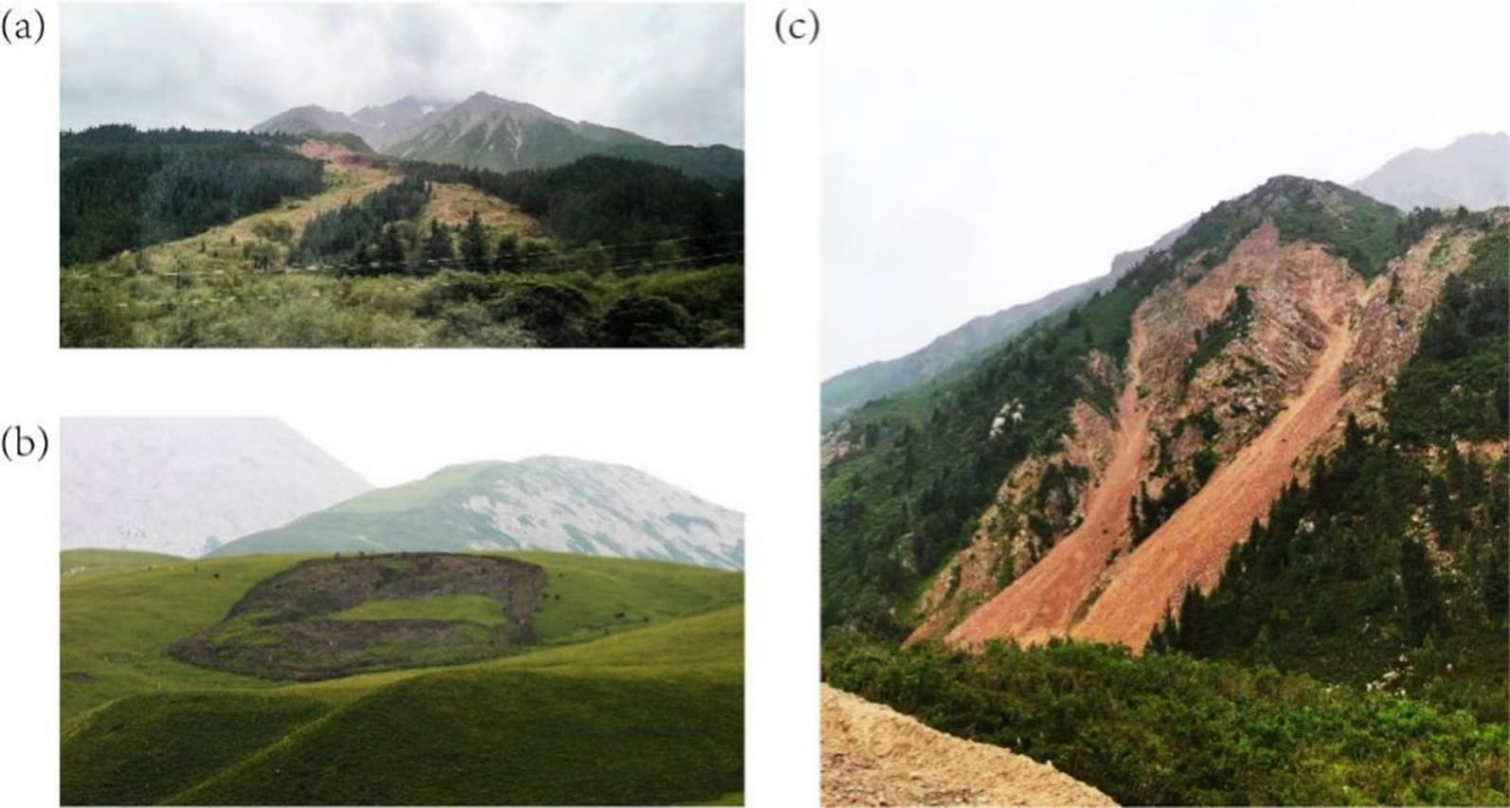
The typical landslide sites on the Qinghai-Tibet Plateau during the field trip. **a** A landslide site distinguished by the surface vegetation, **b** A typical circular arc landslide with a relatively intact landslide bench, **c** A debris slide site with short sliding distance.

**12:**
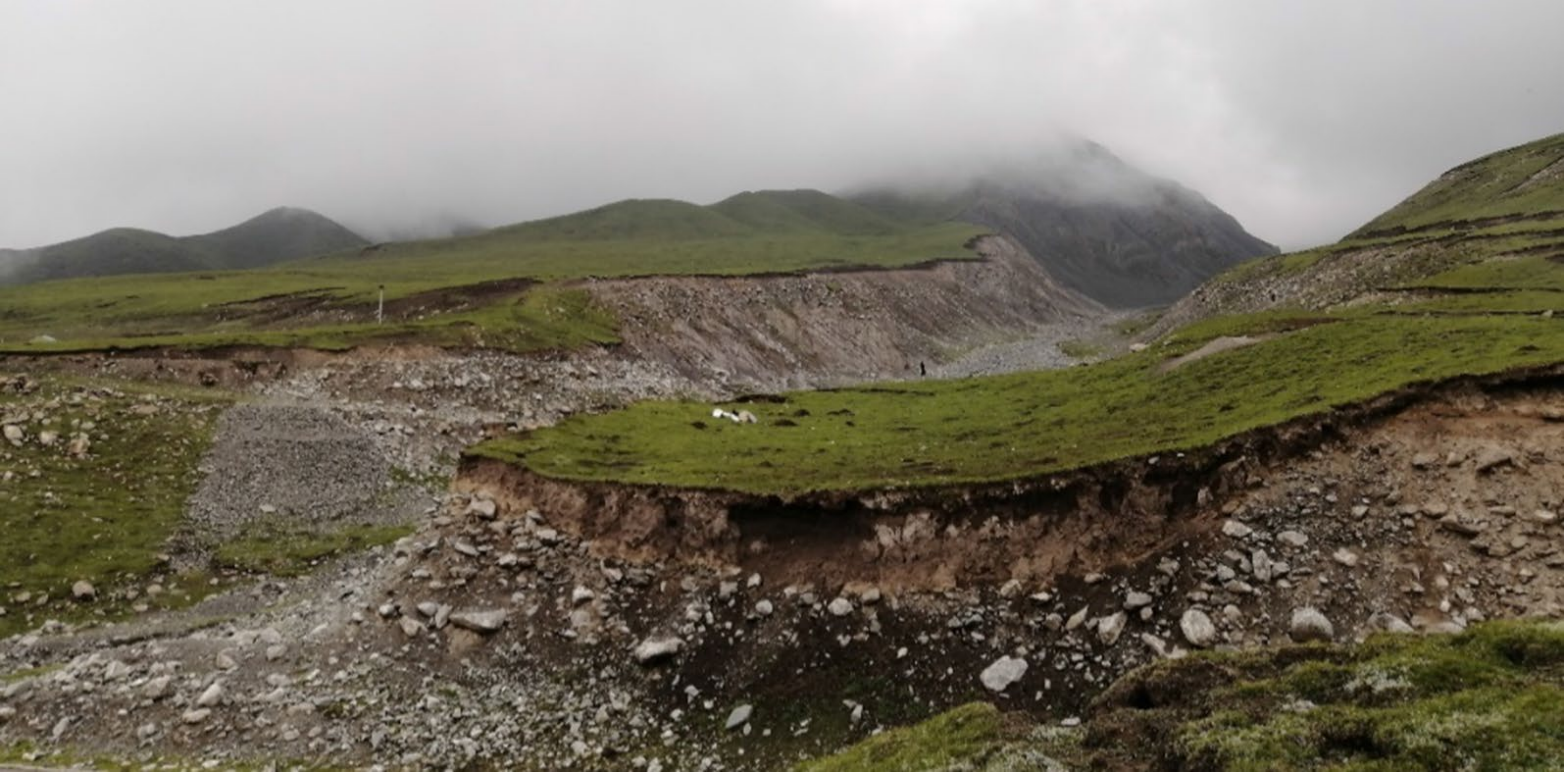
A debris flow site on the Qinghai-Tibet Plateau found during the field trip

At the end of the field trip, the group passed through the Riyue Mountain, which is almost the joint between the Qinghai-Tibet Plateau and the Loess Plateau. At this site, the Loess Plateau landform was immediately characterized by its Cenozoic faulted basins (Fig. 13).

**13:**
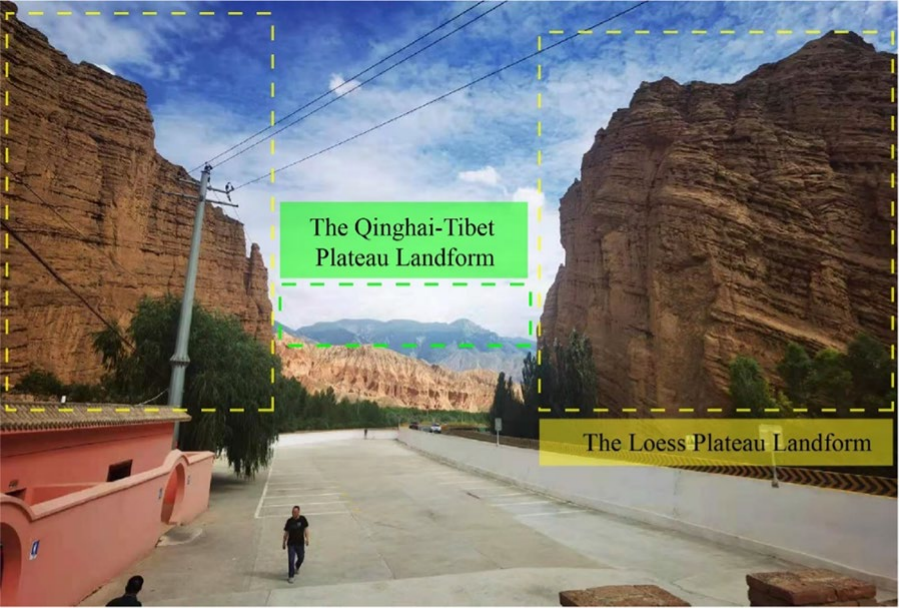
The loess plateau landform to the east of Riyue Mountain in Qinghai Province

Qinghai Province is the third largest province in China with an area of 0.72 million km^2^. It is a huge Geology and Geo-disaster museum. This trip achieved the aims to let more scholars and Geo-disaster engineers know about Qinghai, and to deepen and apply basic researches on the main issues related to plateau science, environment protection and sustainable economic and social development in Plateau areas.

(Reported by Fenggui Liu (Qinghai Normal University, China), Guolong Zhu (Tongji University, China) and Fawu Wang (Tongji University, China).

